# Crystal structure of (2-{3-[4-(4′-cyano­biphenyl-4-yl­oxy)but­oxy]pyridin-2-yl-κ*N*}-5-(dodec­yloxy)phenyl-κ*C* ^1^)(9-oxo­tetra­cos-7-en-7-olato-κ^2^ *O*,*O*′)platinum(II)

**DOI:** 10.1107/S1600536814021928

**Published:** 2014-11-05

**Authors:** Kaijun Luo, Hanlin Liu, Qing Guo, Daibing Luo

**Affiliations:** a College of Chemistry and Materials Science, Sichuan Normal University, Chengdu, Sichuan 610068, People’s Republic of China; b Analytical and Testing Center, Sichuan University, Chengdu, Sichuan 610065, People’s Republic of China

**Keywords:** crystal structure, platinum(II) complex, 9-oxo­tetra­cos-7-en-7-olate

## Abstract

The central Pt^II^ atom in the title compound, [Pt(C_40_H_47_N_2_O_3_)(C_24_H_45_O_2_)], has a slightly distorted square-planar coordination environment. The dihedral angle between the plane formed by Pt and the chelating C and N atoms and that formed by Pt and the chelating O atoms is 2.1 (3)°. The angle between the planes of the two rings in the biphenyl-4-carbo­nitrile unit is 35.1 (5)°. One lateral alkane chain is disordered over two sets of sites with site occupancy factors in a 0.595 (7):0.405 (7) ratio.

## Related literature   

For general background to related structures, see: Yang & Hsu (2009 ▶); Lamansky *et al.* (2001 ▶); Brooks *et al.* (2002 ▶); Wang *et al.* (2011 ▶, 2012 ▶). For the synthesis of some precursor products, see: Jiang *et al.* (2011 ▶).
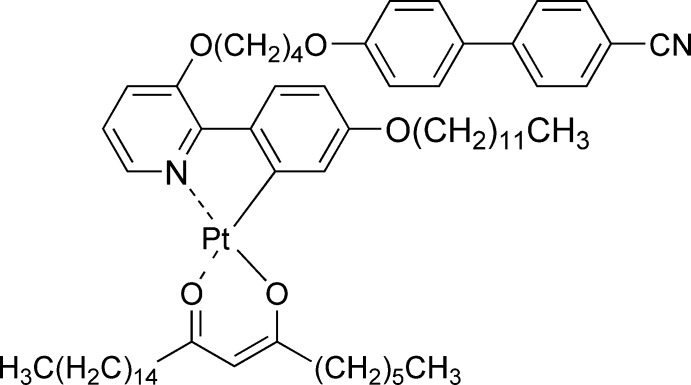


## Experimental   

### Crystal data  

[Pt(C_40_H_47_N_2_O_3_)(C_24_H_45_O_2_)]
*M* *_r_* = 1164.49
Triclinic, 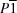

*a* = 8.4938 (3) Å
*b* = 18.9062 (7) Å
*c* = 19.9303 (7) Å
α = 88.215 (3)°
β = 78.816 (3)°
γ = 83.072 (3)°
*V* = 3116.71 (19) Å^3^
*Z* = 2
Mo *K*α radiation
μ = 2.30 mm^−1^
*T* = 293 K
0.23 × 0.20 × 0.07 mm

### Data collection  

Oxford Diffraction Xcalibur, Eos diffractometer
Absorption correction: multi-scan (*CrysAlis PRO*; Oxford Diffraction, 2007 ▶) *T*_min_ = 0.620, *T*_max_ = 0.856
25616 measured reflections
10933 independent reflections
7310 reflections with *I* > 2σ(*I*)
*R*_int_ = 0.044

### Refinement  

*R*[*F* ^2^ > 2σ(*F* ^2^)] = 0.061
*wR*(*F* ^2^) = 0.152
*S* = 1.03
10933 reflections
609 parameters
50 restraints
H-atom parameters constrained
Δρ_max_ = 1.42 e Å^−3^
Δρ_min_ = −0.59 e Å^−3^

### 

Data collection: *CrysAlis PRO* (Oxford Diffraction, 2007 ▶); cell refinement: *CrysAlis PRO*; data reduction: *CrysAlis PRO*; program(s) used to solve structure: *SHELXS97* (Sheldrick, 2008 ▶); program(s) used to refine structure: *SHELXL* (Sheldrick, 2008 ▶); molecular graphics: *OLEX2* (Dolomanov *et al.*, 2009 ▶); software used to prepare material for publication: *OLEX2*.

## Supplementary Material

Crystal structure: contains datablock(s) global, I. DOI: 10.1107/S1600536814021928/vn2086sup1.cif

Structure factors: contains datablock(s) I. DOI: 10.1107/S1600536814021928/vn2086Isup2.hkl

**Fig. 1.**
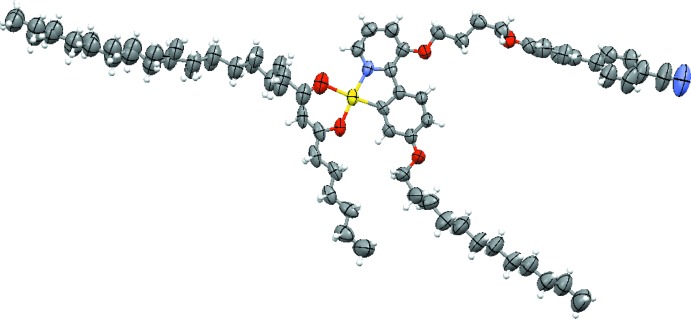
The molecular structure of the title complex, with 50% probability displacement ellipsoids.

. DOI: 10.1107/S1600536814021928/vn2086fig1.tif
The mol­ecular structure of the title complex, with 50% probability displacement ellipsoids.

CCDC reference: 1027628

Additional supporting information:  crystallographic information; 3D view; checkCIF report

## supplementary crystallographic information

### S1. Experimental

2-Phenylpyridine derivative, 4'-(4-(2-(4-(dodecyloxy)phenyl)pyridin-3-yloxy)butoxy)biphenyl-4-carbonitrile (cnbp-Hyyp) and hexacosane-7,9-dione were prepared according to the method of Jiang *et al.* (2011). A mixture of cnbp-Hyyp (1.20 g, 1.6 mmol) and K_2_PtCl_4_ salt (0.58 g,1.6 mmol) was stirred for 12 h at 80 °C in a 3:1 mixture solvent of 2-ethoxyethanol (27 ml) and water (9 ml) under inert atmosphere. After cooled down to room temperature, 10 ml water was added into the reaction mixture. The Pt(II) µ-dichloro-bridged dimer was isolated in water and dried under vacuo at 80 °C. A mixture of the Pt(II) µ-dichloro-bridged dimer (1.18 g, 1.1 mmol), hexacosane-7,9-dione (1.23 g, 3.1 mmol), caesium carbonate (0.15 g, 0.8 mmol) and 2-ethoxyethanol (20 ml) was stirred for 40 min at 90 °C. After cooled down to room temperature, 12 ml ethanol was added into the reaction mixture. The residue was filtered and washed with water. The coarse product was purified through a flash silica gel column using ethyl acetate: petroleum ether (1:1 *v*/*v*) as the eluent to give a light yellow solid (0.47 g, 21.9%. T_m_ = 94 °C). Single crystals of the title complex were obtained by slow diffusion of methanol into the dichloromethane solution of the title complex.

### S2. Refinement

All non-H atoms were refined anisotropically. The H atoms were placed in calculated positions and included in the refinement in a riding mode.

### Crystal data

**Table:**

[Pt(C_40_H_47_N_2_O_3_)(C_24_H_45_O_2_)]	*Z* = 2
*M**_r_* = 1164.49	*F*(000) = 1216
Triclinic, *P*1	*D*_x_ = 1.241 Mg m^−^^3^
Hall symbol: -P 1	Mo *K*α radiation, λ = 0.7107 Å
*a* = 8.4938 (3) Å	Cell parameters from 6800 reflections
*b* = 18.9062 (7) Å	θ = 2.9–29.1°
*c* = 19.9303 (7) Å	µ = 2.30 mm^−^^1^
α = 88.215 (3)°	*T* = 293 K
β = 78.816 (3)°	Plate, yellow
γ = 83.072 (3)°	0.23 × 0.20 × 0.07 mm
*V* = 3116.71 (19) Å^3^	

### Data collection

**Table:**

Oxford Diffraction Xcalibur, Eos diffractometer	10933 independent reflections
Radiation source: Enhance (Mo) X-ray Source	7310 reflections with *I* > 2σ(*I*)
Graphite monochromator	*R*_int_ = 0.044
Detector resolution: 16.0874 pixels mm^-1^	θ_max_ = 25.0°, θ_min_ = 3.1°
ω scans	*h* = −9→10
Absorption correction: multi-scan (*CrysAlis PRO*; Oxford Diffraction, 2007)	*k* = −22→22
*T*_min_ = 0.620, *T*_max_ = 0.856	*l* = 0→23
25616 measured reflections	

### Refinement

**Table:**

Refinement on *F*^2^	50 restraints
Least-squares matrix: full	H-atom parameters constrained
*R*[*F*^2^ > 2σ(*F*^2^)] = 0.061	*w* = 1/[σ^2^(*F*_o_^2^) + (0.0717*P*)^2^] where *P* = (*F*_o_^2^ + 2*F*_c_^2^)/3
*wR*(*F*^2^) = 0.152	(Δ/σ)_max_ = 0.002
*S* = 1.03	Δρ_max_ = 1.42 e Å^−^^3^
10933 reflections	Δρ_min_ = −0.59 e Å^−^^3^
609 parameters	

### Special details

**Table:**

Geometry. All e.s.d.'s (except the e.s.d. in the dihedral angle between two l.s. planes) are estimated using the full covariance matrix. The cell e.s.d.'s are taken into account individually in the estimation of e.s.d.'s in distances, angles and torsion angles; correlations between e.s.d.'s in cell parameters are only used when they are defined by crystal symmetry. An approximate (isotropic) treatment of cell e.s.d.'s is used for estimating e.s.d.'s involving l.s. planes.
Refinement. Refinement of *F*^2^ against ALL reflections. The weighted *R*-factor *wR* and goodness of fit *S* are based on *F*^2^, conventional *R*-factors *R* are based on *F*, with *F* set to zero for negative *F*^2^. The threshold expression of *F*^2^ > σ(*F*^2^) is used only for calculating *R*-factors(gt) *etc*. and is not relevant to the choice of reflections for refinement. *R*-factors based on *F*^2^ are statistically about twice as large as those based on *F*, and *R*- factors based on ALL data will be even larger.Moreover, some of C–C bond length for disordered lateral alkane chain and atomic thermal vibration parameter, which affect refinement procedure, were restrained.

### Fractional atomic coordinates and isotropic or equivalent isotropic displacement parameters (Å^2^)

**Table:**

	*x*	*y*	*z*	*U*_iso_*/*U*_eq_	Occ. (<1)
Pt1	0.24385 (3)	0.104200 (16)	0.880858 (12)	0.07868 (15)	
O1	0.0386 (6)	0.0611 (3)	0.9070 (2)	0.0864 (14)	
O2	0.1414 (9)	0.1965 (4)	0.9350 (3)	0.1312 (14)	
O3	0.8301 (6)	0.0681 (3)	0.7414 (2)	0.0847 (13)	
O4	1.2933 (6)	−0.0392 (3)	0.5417 (3)	0.1008 (16)	
O5	0.3111 (6)	−0.1570 (3)	0.7671 (3)	0.1007 (16)	
N1	0.4586 (7)	0.1377 (3)	0.8511 (3)	0.0708 (15)	
N2	1.969 (2)	−0.4363 (9)	0.1741 (7)	0.260 (8)	
C1	0.5707 (8)	0.0925 (4)	0.8084 (3)	0.0685 (18)	
C2	0.7280 (9)	0.1124 (4)	0.7858 (3)	0.0729 (19)	
C3	0.7677 (10)	0.1755 (5)	0.8086 (4)	0.090 (2)	
H3	0.8710	0.1888	0.7945	0.108*	
C4	0.6523 (12)	0.2177 (5)	0.8523 (4)	0.093 (2)	
H4	0.6775	0.2597	0.8686	0.111*	
C5	0.5027 (11)	0.1986 (4)	0.8716 (4)	0.085 (2)	
H5	0.4259	0.2287	0.9005	0.102*	
C6	0.5083 (8)	0.0258 (4)	0.7930 (3)	0.0665 (17)	
C7	0.5978 (8)	−0.0286 (4)	0.7512 (3)	0.0733 (18)	
H7	0.7029	−0.0244	0.7286	0.088*	
C8	0.5254 (9)	−0.0885 (4)	0.7445 (4)	0.083 (2)	
H8	0.5830	−0.1253	0.7168	0.100*	
C9	0.3678 (9)	−0.0957 (5)	0.7782 (4)	0.082 (2)	
C10	0.2801 (8)	−0.0411 (4)	0.8183 (3)	0.0727 (18)	
H10	0.1741	−0.0451	0.8398	0.087*	
C11	0.3515 (7)	0.0216 (4)	0.8270 (3)	0.0675 (18)	
C12	0.9947 (9)	0.0833 (5)	0.7188 (4)	0.088 (2)	
H12A	1.0487	0.0844	0.7574	0.106*	
H12B	0.9976	0.1290	0.6952	0.106*	
C13	1.0748 (9)	0.0232 (5)	0.6706 (4)	0.091 (2)	
H13A	1.0194	0.0236	0.6324	0.109*	
H13B	1.0639	−0.0219	0.6946	0.109*	
C14	1.2529 (9)	0.0289 (5)	0.6435 (4)	0.099 (3)	
H14A	1.2627	0.0693	0.6125	0.119*	
H14B	1.3040	0.0377	0.6815	0.119*	
C15	1.3406 (10)	−0.0368 (5)	0.6068 (4)	0.101 (3)	
H15A	1.4564	−0.0356	0.6004	0.121*	
H15B	1.3137	−0.0790	0.6335	0.121*	
C16	1.3701 (9)	−0.0925 (5)	0.4992 (4)	0.092 (2)	
C17	1.4668 (11)	−0.1505 (5)	0.5184 (4)	0.108 (3)	
H17	1.4813	−0.1558	0.5634	0.129*	
C18	1.5423 (11)	−0.2008 (5)	0.4700 (4)	0.111 (3)	
H18	1.6028	−0.2409	0.4840	0.133*	
C19	1.5316 (10)	−0.1939 (5)	0.4021 (4)	0.092 (2)	
C20	1.4322 (10)	−0.1347 (5)	0.3843 (4)	0.096 (2)	
H20	1.4198	−0.1294	0.3390	0.115*	
C21	1.3527 (9)	−0.0845 (5)	0.4303 (4)	0.092 (2)	
H21	1.2882	−0.0456	0.4165	0.110*	
C22	1.6224 (11)	−0.2455 (5)	0.3514 (4)	0.101 (3)	
C23	1.6491 (16)	−0.3181 (6)	0.3678 (5)	0.148 (4)	
H23	1.6043	−0.3337	0.4113	0.178*	
C24	1.7386 (19)	−0.3664 (7)	0.3222 (7)	0.181 (6)	
H24	1.7565	−0.4140	0.3348	0.217*	
C25	1.8031 (17)	−0.3439 (7)	0.2565 (6)	0.151 (4)	
C26	1.7752 (12)	−0.2741 (6)	0.2373 (5)	0.118 (3)	
H26	1.8153	−0.2594	0.1930	0.141*	
C27	1.6871 (11)	−0.2262 (6)	0.2845 (4)	0.102 (3)	
H27	1.6697	−0.1787	0.2713	0.122*	
C28	1.892 (2)	−0.3965 (8)	0.2113 (7)	0.196 (7)	
C29	0.1523 (10)	−0.1683 (5)	0.8017 (4)	0.105 (3)	
H29A	0.0721	−0.1355	0.7845	0.126*	
H29B	0.1408	−0.1607	0.8504	0.126*	
C30	0.1290 (13)	−0.2456 (6)	0.7882 (5)	0.139 (4)	
H30A	0.2150	−0.2776	0.8022	0.167*	
H30B	0.0272	−0.2568	0.8154	0.167*	
C31	0.1294 (14)	−0.2569 (6)	0.7170 (5)	0.146 (4)	
H31A	0.2213	−0.2376	0.6890	0.175*	
H31B	0.0317	−0.2321	0.7049	0.175*	
C32	0.1388 (16)	−0.3392 (6)	0.7021 (6)	0.154 (4)	
H32A	0.2264	−0.3645	0.7212	0.185*	
H32B	0.0391	−0.3564	0.7251	0.185*	
C33	0.1643 (17)	−0.3555 (6)	0.6299 (6)	0.166 (5)	
H33A	0.2596	−0.3346	0.6071	0.199*	
H33B	0.0732	−0.3317	0.6121	0.199*	
C34	0.1852 (16)	−0.4328 (5)	0.6100 (6)	0.146 (4)	
H34A	0.2724	−0.4572	0.6300	0.175*	
H34B	0.0873	−0.4530	0.6305	0.175*	
C35	0.2200 (17)	−0.4484 (6)	0.5363 (6)	0.156 (4)	
H35A	0.1303	−0.4252	0.5171	0.188*	
H35B	0.3145	−0.4256	0.5158	0.188*	
C36	0.2483 (15)	−0.5221 (6)	0.5139 (5)	0.139 (4)	
H36A	0.1546	−0.5450	0.5350	0.167*	
H36B	0.3392	−0.5450	0.5325	0.167*	
C37	0.2809 (16)	−0.5378 (6)	0.4398 (6)	0.153 (4)	
H37A	0.1893	−0.5161	0.4210	0.184*	
H37B	0.3740	−0.5147	0.4182	0.184*	
C38	0.3112 (17)	−0.6134 (6)	0.4204 (6)	0.156 (4)	
H38A	0.2170	−0.6363	0.4409	0.187*	
H38B	0.4012	−0.6354	0.4401	0.187*	
C39	0.3474 (17)	−0.6282 (7)	0.3463 (6)	0.171 (5)	
H39A	0.2584	−0.6054	0.3264	0.205*	
H39B	0.4429	−0.6061	0.3259	0.205*	
C40	0.375 (2)	−0.7038 (8)	0.3270 (7)	0.215 (7)	
H40A	0.4323	−0.7084	0.2806	0.323*	
H40B	0.2724	−0.7220	0.3310	0.323*	
H40C	0.4367	−0.7302	0.3568	0.323*	
C41	−0.0896 (9)	0.0900 (5)	0.9480 (4)	0.091 (3)	
C42	−0.1078 (11)	0.1551 (6)	0.9779 (4)	0.111 (3)	
H42	−0.2066	0.1676	1.0068	0.133*	
C43	0.0003 (16)	0.2050 (6)	0.9712 (6)	0.1312 (14)	
C44	−0.2244 (10)	0.0416 (6)	0.9605 (4)	0.107 (3)	
H44A	−0.2503	0.0317	1.0092	0.129*	
H44B	−0.3198	0.0677	0.9478	0.129*	
C45	−0.1905 (9)	−0.0283 (5)	0.9235 (4)	0.096 (3)	
H45A	−0.0971	−0.0556	0.9368	0.115*	
H45B	−0.1641	−0.0192	0.8747	0.115*	
C46	−0.3305 (9)	−0.0718 (5)	0.9381 (4)	0.099 (3)	
H46A	−0.3608	−0.0786	0.9871	0.118*	
H46B	−0.4221	−0.0454	0.9225	0.118*	
C47	−0.2946 (10)	−0.1431 (6)	0.9044 (4)	0.113 (3)	
H47A	−0.2639	−0.1358	0.8555	0.136*	
H47B	−0.2020	−0.1688	0.9199	0.136*	
C48	−0.4285 (13)	−0.1890 (7)	0.9171 (5)	0.134 (4)	
H48A	−0.4585	−0.1968	0.9660	0.161*	
H48B	−0.5215	−0.1632	0.9021	0.161*	
C49	−0.3936 (17)	−0.2591 (8)	0.8834 (7)	0.193 (6)	
H49A	−0.3858	−0.2526	0.8350	0.289*	
H49B	−0.4790	−0.2876	0.9009	0.289*	
H49C	−0.2933	−0.2827	0.8925	0.289*	
C50	−0.025 (2)	0.2803 (9)	1.0074 (10)	0.1312 (14)	0.595 (7)
H50A	−0.0691	0.2755	1.0558	0.157*	0.595 (7)
H50B	0.0769	0.2999	1.0023	0.157*	0.595 (7)
C62	−1.009 (4)	0.8053 (15)	1.4640 (12)	0.151 (2)	0.595 (7)
H62A	−1.1167	0.8057	1.4545	0.181*	0.595 (7)
H62B	−0.9582	0.8427	1.4367	0.181*	0.595 (7)
C60	−0.878 (4)	0.7127 (12)	1.3689 (13)	0.151 (2)	0.595 (7)
H60A	−0.8091	0.7465	1.3451	0.181*	0.595 (7)
H60B	−0.9805	0.7229	1.3538	0.181*	0.595 (7)
C61	−0.912 (4)	0.7339 (14)	1.4424 (13)	0.151 (2)	0.595 (7)
H61A	−0.8091	0.7329	1.4567	0.181*	0.595 (7)
H61B	−0.9686	0.6972	1.4685	0.181*	0.595 (7)
C64	−1.113 (3)	0.8968 (10)	1.5474 (10)	0.151 (2)	0.595 (7)
H64A	−1.1772	0.9052	1.5128	0.226*	0.595 (7)
H64B	−1.0365	0.9310	1.5427	0.226*	0.595 (7)
H64C	−1.1816	0.9012	1.5918	0.226*	0.595 (7)
C63	−1.022 (3)	0.8213 (11)	1.5396 (11)	0.151 (2)	0.595 (7)
H63A	−0.9155	0.8201	1.5511	0.181*	0.595 (7)
H63B	−1.0815	0.7875	1.5685	0.181*	0.595 (7)
C58	−0.686 (3)	0.6430 (11)	1.2697 (12)	0.151 (2)	0.595 (7)
H58A	−0.5852	0.6593	1.2756	0.181*	0.595 (7)
H58B	−0.7318	0.6740	1.2370	0.181*	0.595 (7)
C59	−0.806 (3)	0.6415 (12)	1.3394 (11)	0.151 (2)	0.595 (7)
H59A	−0.8935	0.6150	1.3334	0.181*	0.595 (7)
H59B	−0.7505	0.6155	1.3723	0.181*	0.595 (7)
C56	−0.559 (2)	0.5630 (9)	1.1725 (8)	0.1312 (14)	0.595 (7)
H56A	−0.5023	0.6050	1.1642	0.157*	0.595 (7)
H56B	−0.6302	0.5632	1.1399	0.157*	0.595 (7)
C57	−0.657 (2)	0.5640 (10)	1.2453 (10)	0.151 (2)	0.595 (7)
H57A	−0.5979	0.5345	1.2751	0.181*	0.595 (7)
H57B	−0.7591	0.5459	1.2463	0.181*	0.595 (7)
C51	−0.144 (2)	0.3293 (8)	0.9724 (8)	0.1312 (14)	0.595 (7)
H51A	−0.2483	0.3112	0.9824	0.157*	0.595 (7)
H51B	−0.1059	0.3268	0.9233	0.157*	0.595 (7)
C52	−0.168 (2)	0.4101 (8)	0.9945 (7)	0.1312 (14)	0.595 (7)
H52A	−0.0654	0.4293	0.9884	0.157*	0.595 (7)
H52B	−0.2379	0.4388	0.9683	0.157*	0.595 (7)
C54	−0.316 (2)	0.4857 (8)	1.0949 (8)	0.1312 (14)	0.595 (7)
H54A	−0.2255	0.5118	1.0971	0.157*	0.595 (7)
H54B	−0.3719	0.5090	1.0606	0.157*	0.595 (7)
C53	−0.247 (2)	0.4074 (8)	1.0701 (8)	0.1312 (14)	0.595 (7)
H53A	−0.1683	0.3873	1.0968	0.157*	0.595 (7)
H53B	−0.3342	0.3775	1.0764	0.157*	0.595 (7)
C55	−0.429 (2)	0.4915 (9)	1.1628 (8)	0.1312 (14)	0.595 (7)
H55A	−0.3657	0.4902	1.1985	0.157*	0.595 (7)
H55B	−0.4882	0.4502	1.1691	0.157*	0.595 (7)
C50A	−0.079 (3)	0.2691 (11)	1.0120 (13)	0.1312 (14)	0.405 (7)
H50C	−0.0479	0.2672	1.0565	0.157*	0.405 (7)
H50D	−0.1952	0.2711	1.0187	0.157*	0.405 (7)
C51A	−0.023 (4)	0.3336 (11)	0.9727 (12)	0.1312 (14)	0.405 (7)
H51C	−0.0626	0.3389	0.9301	0.157*	0.405 (7)
H51D	0.0940	0.3305	0.9628	0.157*	0.405 (7)
C52A	−0.099 (3)	0.3978 (13)	1.0228 (13)	0.1312 (14)	0.405 (7)
H52C	−0.0362	0.4372	1.0094	0.157*	0.405 (7)
H52D	−0.0833	0.3832	1.0684	0.157*	0.405 (7)
C53A	−0.290 (3)	0.4289 (12)	1.0289 (14)	0.1312 (14)	0.405 (7)
H53C	−0.3097	0.4271	0.9827	0.157*	0.405 (7)
H53D	−0.3489	0.3929	1.0547	0.157*	0.405 (7)
C54A	−0.383 (3)	0.5025 (12)	1.0582 (11)	0.1312 (14)	0.405 (7)
H54C	−0.3036	0.5350	1.0590	0.157*	0.405 (7)
H54D	−0.4499	0.5219	1.0261	0.157*	0.405 (7)
C55A	−0.491 (3)	0.5024 (13)	1.1296 (11)	0.1312 (14)	0.405 (7)
H55C	−0.4704	0.4561	1.1504	0.157*	0.405 (7)
H55D	−0.6030	0.5078	1.1238	0.157*	0.405 (7)
C56A	−0.473 (3)	0.5612 (14)	1.1816 (12)	0.1312 (14)	0.405 (7)
H56C	−0.3649	0.5510	1.1909	0.157*	0.405 (7)
H56D	−0.4763	0.6062	1.1568	0.157*	0.405 (7)
C57A	−0.585 (3)	0.5752 (16)	1.2507 (14)	0.151 (2)	0.405 (7)
H57C	−0.5130	0.5803	1.2820	0.181*	0.405 (7)
H57D	−0.6284	0.5303	1.2630	0.181*	0.405 (7)
C58A	−0.732 (4)	0.6331 (17)	1.2738 (15)	0.151 (2)	0.405 (7)
H58C	−0.7159	0.6746	1.2445	0.181*	0.405 (7)
H58D	−0.8284	0.6149	1.2657	0.181*	0.405 (7)
C59A	−0.763 (4)	0.6576 (17)	1.3484 (14)	0.151 (2)	0.405 (7)
H59C	−0.8154	0.6217	1.3775	0.181*	0.405 (7)
H59D	−0.6606	0.6613	1.3616	0.181*	0.405 (7)
C60A	−0.869 (6)	0.7290 (19)	1.361 (2)	0.151 (2)	0.405 (7)
H60C	−0.8034	0.7680	1.3508	0.181*	0.405 (7)
H60D	−0.9501	0.7338	1.3327	0.181*	0.405 (7)
C61A	−0.947 (5)	0.729 (2)	1.4362 (18)	0.151 (2)	0.405 (7)
H61C	−0.8758	0.7016	1.4626	0.181*	0.405 (7)
H61D	−1.0475	0.7075	1.4426	0.181*	0.405 (7)
C62A	−0.980 (6)	0.807 (2)	1.4593 (17)	0.151 (2)	0.405 (7)
H62C	−0.8788	0.8266	1.4555	0.181*	0.405 (7)
H62D	−1.0440	0.8343	1.4300	0.181*	0.405 (7)
C63A	−1.072 (4)	0.8108 (17)	1.5336 (14)	0.151 (2)	0.405 (7)
H63C	−1.0124	0.7752	1.5583	0.181*	0.405 (7)
H63D	−1.1742	0.7934	1.5331	0.181*	0.405 (7)
C64A	−1.113 (4)	0.8766 (15)	1.5802 (14)	0.151 (2)	0.405 (7)
H64D	−1.0301	0.9076	1.5683	0.226*	0.405 (7)
H64E	−1.1196	0.8616	1.6271	0.226*	0.405 (7)
H64F	−1.2148	0.9015	1.5744	0.226*	0.405 (7)

### Atomic displacement parameters (Å^2^)

**Table:**

	*U*^11^	*U*^22^	*U*^33^	*U*^12^	*U*^13^	*U*^23^
Pt1	0.0873 (2)	0.0880 (3)	0.05385 (18)	0.01122 (16)	−0.00836 (13)	−0.01002 (14)
O1	0.089 (3)	0.089 (4)	0.067 (3)	0.027 (3)	−0.003 (2)	−0.010 (3)
O2	0.146 (4)	0.122 (3)	0.115 (3)	0.033 (3)	−0.024 (2)	−0.028 (2)
O3	0.073 (3)	0.095 (4)	0.082 (3)	−0.009 (3)	−0.003 (2)	−0.013 (3)
O4	0.094 (4)	0.125 (5)	0.079 (3)	0.006 (3)	−0.019 (3)	−0.006 (3)
O5	0.092 (4)	0.087 (4)	0.115 (4)	−0.020 (3)	0.010 (3)	−0.029 (3)
N1	0.085 (4)	0.070 (4)	0.055 (3)	0.012 (3)	−0.019 (3)	−0.013 (3)
N2	0.317 (19)	0.246 (17)	0.179 (12)	0.085 (14)	−0.014 (12)	−0.079 (12)
C1	0.066 (4)	0.093 (6)	0.048 (3)	0.002 (4)	−0.020 (3)	−0.006 (3)
C2	0.090 (5)	0.069 (5)	0.061 (4)	0.000 (4)	−0.022 (4)	−0.002 (3)
C3	0.103 (6)	0.091 (6)	0.074 (5)	−0.010 (5)	−0.015 (4)	−0.007 (4)
C4	0.115 (7)	0.085 (6)	0.086 (5)	−0.015 (5)	−0.037 (5)	−0.007 (4)
C5	0.103 (6)	0.086 (6)	0.067 (4)	0.005 (5)	−0.024 (4)	−0.015 (4)
C6	0.069 (4)	0.074 (5)	0.055 (4)	−0.001 (4)	−0.012 (3)	−0.002 (3)
C7	0.062 (4)	0.081 (5)	0.073 (4)	0.001 (4)	−0.006 (3)	−0.014 (4)
C8	0.076 (5)	0.093 (6)	0.075 (5)	0.001 (4)	−0.004 (4)	−0.028 (4)
C9	0.080 (5)	0.088 (6)	0.072 (4)	−0.005 (4)	0.001 (4)	−0.013 (4)
C10	0.064 (4)	0.084 (5)	0.066 (4)	−0.009 (4)	−0.004 (3)	0.000 (4)
C11	0.045 (3)	0.100 (6)	0.053 (4)	0.001 (3)	−0.005 (3)	0.011 (3)
C12	0.079 (5)	0.108 (7)	0.076 (5)	−0.013 (4)	−0.008 (4)	0.005 (4)
C13	0.075 (5)	0.107 (7)	0.089 (5)	−0.010 (4)	−0.013 (4)	−0.001 (5)
C14	0.092 (6)	0.123 (8)	0.085 (5)	−0.025 (5)	−0.015 (4)	0.001 (5)
C15	0.095 (6)	0.133 (8)	0.072 (5)	−0.010 (5)	−0.011 (4)	−0.009 (5)
C16	0.080 (5)	0.114 (7)	0.081 (5)	−0.006 (5)	−0.012 (4)	−0.001 (5)
C17	0.126 (7)	0.120 (8)	0.076 (5)	0.004 (6)	−0.027 (5)	0.010 (5)
C18	0.144 (8)	0.105 (7)	0.076 (5)	0.022 (6)	−0.026 (5)	0.006 (5)
C19	0.107 (6)	0.097 (6)	0.074 (5)	−0.013 (5)	−0.024 (4)	0.014 (5)
C20	0.102 (6)	0.118 (7)	0.070 (5)	−0.011 (5)	−0.023 (4)	0.010 (5)
C21	0.085 (5)	0.108 (7)	0.082 (5)	−0.012 (5)	−0.017 (4)	0.011 (5)
C22	0.127 (7)	0.092 (7)	0.081 (6)	0.002 (5)	−0.022 (5)	0.006 (5)
C23	0.226 (12)	0.110 (9)	0.091 (7)	0.017 (8)	−0.012 (7)	0.008 (7)
C24	0.292 (17)	0.112 (10)	0.116 (9)	0.034 (10)	−0.023 (10)	−0.001 (8)
C25	0.213 (13)	0.133 (11)	0.089 (8)	0.026 (9)	−0.012 (7)	−0.021 (7)
C26	0.137 (8)	0.135 (10)	0.071 (6)	0.009 (7)	−0.011 (5)	−0.008 (6)
C27	0.114 (7)	0.124 (8)	0.070 (5)	−0.016 (6)	−0.024 (5)	0.014 (5)
C28	0.288 (19)	0.152 (13)	0.118 (10)	0.068 (12)	−0.015 (10)	−0.040 (9)
C29	0.101 (6)	0.111 (7)	0.101 (6)	−0.033 (5)	0.005 (5)	−0.021 (5)
C30	0.140 (9)	0.156 (10)	0.117 (8)	−0.058 (8)	0.014 (6)	−0.038 (7)
C31	0.172 (10)	0.154 (11)	0.125 (8)	−0.068 (8)	−0.031 (7)	−0.016 (7)
C32	0.203 (12)	0.128 (10)	0.140 (9)	−0.020 (8)	−0.049 (8)	−0.042 (8)
C33	0.206 (13)	0.119 (10)	0.169 (11)	−0.024 (9)	−0.021 (9)	−0.049 (8)
C34	0.209 (12)	0.088 (7)	0.142 (9)	−0.002 (7)	−0.042 (8)	−0.027 (7)
C35	0.215 (13)	0.099 (9)	0.151 (11)	−0.012 (8)	−0.023 (9)	−0.038 (7)
C36	0.208 (12)	0.090 (8)	0.122 (8)	−0.015 (7)	−0.040 (8)	−0.006 (6)
C37	0.229 (14)	0.108 (9)	0.115 (9)	−0.022 (9)	−0.008 (8)	−0.020 (7)
C38	0.217 (13)	0.125 (10)	0.124 (9)	−0.032 (9)	−0.019 (8)	−0.023 (7)
C39	0.224 (14)	0.142 (11)	0.134 (10)	−0.005 (10)	−0.005 (9)	−0.038 (8)
C40	0.278 (18)	0.184 (15)	0.170 (12)	−0.041 (13)	0.009 (11)	−0.066 (11)
C41	0.076 (5)	0.130 (8)	0.054 (4)	0.023 (5)	−0.001 (4)	0.009 (5)
C42	0.109 (6)	0.142 (8)	0.062 (5)	0.057 (4)	−0.006 (4)	−0.028 (5)
C43	0.146 (4)	0.122 (3)	0.115 (3)	0.033 (3)	−0.024 (2)	−0.028 (2)
C44	0.088 (6)	0.154 (9)	0.060 (5)	0.038 (6)	0.002 (4)	0.010 (5)
C45	0.061 (5)	0.145 (8)	0.070 (5)	0.013 (5)	−0.003 (4)	0.014 (5)
C46	0.084 (6)	0.141 (8)	0.063 (4)	0.009 (5)	−0.010 (4)	0.012 (5)
C47	0.083 (6)	0.177 (11)	0.086 (6)	−0.037 (7)	−0.026 (5)	0.026 (6)
C48	0.120 (8)	0.184 (12)	0.103 (7)	−0.045 (8)	−0.019 (6)	0.005 (8)
C49	0.181 (13)	0.203 (15)	0.197 (13)	−0.079 (12)	−0.004 (10)	−0.031 (12)
C50	0.146 (4)	0.122 (3)	0.115 (3)	0.033 (3)	−0.024 (2)	−0.028 (2)
C62	0.171 (6)	0.128 (4)	0.140 (4)	−0.007 (4)	−0.006 (4)	−0.002 (4)
C60	0.171 (6)	0.128 (4)	0.140 (4)	−0.007 (4)	−0.006 (4)	−0.002 (4)
C61	0.171 (6)	0.128 (4)	0.140 (4)	−0.007 (4)	−0.006 (4)	−0.002 (4)
C64	0.171 (6)	0.128 (4)	0.140 (4)	−0.007 (4)	−0.006 (4)	−0.002 (4)
C63	0.171 (6)	0.128 (4)	0.140 (4)	−0.007 (4)	−0.006 (4)	−0.002 (4)
C58	0.171 (6)	0.128 (4)	0.140 (4)	−0.007 (4)	−0.006 (4)	−0.002 (4)
C59	0.171 (6)	0.128 (4)	0.140 (4)	−0.007 (4)	−0.006 (4)	−0.002 (4)
C56	0.146 (4)	0.122 (3)	0.115 (3)	0.033 (3)	−0.024 (2)	−0.028 (2)
C57	0.171 (6)	0.128 (4)	0.140 (4)	−0.007 (4)	−0.006 (4)	−0.002 (4)
C51	0.146 (4)	0.122 (3)	0.115 (3)	0.033 (3)	−0.024 (2)	−0.028 (2)
C52	0.146 (4)	0.122 (3)	0.115 (3)	0.033 (3)	−0.024 (2)	−0.028 (2)
C54	0.146 (4)	0.122 (3)	0.115 (3)	0.033 (3)	−0.024 (2)	−0.028 (2)
C53	0.146 (4)	0.122 (3)	0.115 (3)	0.033 (3)	−0.024 (2)	−0.028 (2)
C55	0.146 (4)	0.122 (3)	0.115 (3)	0.033 (3)	−0.024 (2)	−0.028 (2)
C50A	0.146 (4)	0.122 (3)	0.115 (3)	0.033 (3)	−0.024 (2)	−0.028 (2)
C51A	0.146 (4)	0.122 (3)	0.115 (3)	0.033 (3)	−0.024 (2)	−0.028 (2)
C52A	0.146 (4)	0.122 (3)	0.115 (3)	0.033 (3)	−0.024 (2)	−0.028 (2)
C53A	0.146 (4)	0.122 (3)	0.115 (3)	0.033 (3)	−0.024 (2)	−0.028 (2)
C54A	0.146 (4)	0.122 (3)	0.115 (3)	0.033 (3)	−0.024 (2)	−0.028 (2)
C55A	0.146 (4)	0.122 (3)	0.115 (3)	0.033 (3)	−0.024 (2)	−0.028 (2)
C56A	0.146 (4)	0.122 (3)	0.115 (3)	0.033 (3)	−0.024 (2)	−0.028 (2)
C57A	0.171 (6)	0.128 (4)	0.140 (4)	−0.007 (4)	−0.006 (4)	−0.002 (4)
C58A	0.171 (6)	0.128 (4)	0.140 (4)	−0.007 (4)	−0.006 (4)	−0.002 (4)
C59A	0.171 (6)	0.128 (4)	0.140 (4)	−0.007 (4)	−0.006 (4)	−0.002 (4)
C60A	0.171 (6)	0.128 (4)	0.140 (4)	−0.007 (4)	−0.006 (4)	−0.002 (4)
C61A	0.171 (6)	0.128 (4)	0.140 (4)	−0.007 (4)	−0.006 (4)	−0.002 (4)
C62A	0.171 (6)	0.128 (4)	0.140 (4)	−0.007 (4)	−0.006 (4)	−0.002 (4)
C63A	0.171 (6)	0.128 (4)	0.140 (4)	−0.007 (4)	−0.006 (4)	−0.002 (4)
C64A	0.171 (6)	0.128 (4)	0.140 (4)	−0.007 (4)	−0.006 (4)	−0.002 (4)

### Geometric parameters (Å, º)

**Table:**

Pt1—O1	1.984 (5)	C44—H44B	0.9700
Pt1—O2	2.086 (7)	C44—C45	1.502 (12)
Pt1—N1	1.979 (6)	C45—H45A	0.9700
Pt1—C11	1.952 (7)	C45—H45B	0.9700
O1—C41	1.301 (8)	C45—C46	1.504 (11)
O2—C43	1.267 (13)	C46—H46A	0.9700
O3—C2	1.346 (8)	C46—H46B	0.9700
O3—C12	1.444 (8)	C46—C47	1.497 (12)
O4—C15	1.435 (8)	C47—H47A	0.9700
O4—C16	1.356 (9)	C47—H47B	0.9700
O5—C9	1.346 (9)	C47—C48	1.491 (12)
O5—C29	1.428 (9)	C48—H48A	0.9700
N1—C1	1.377 (8)	C48—H48B	0.9700
N1—C5	1.351 (9)	C48—C49	1.475 (16)
N2—C28	1.127 (15)	C49—H49A	0.9600
C1—C2	1.416 (9)	C49—H49B	0.9600
C1—C6	1.488 (10)	C49—H49C	0.9600
C2—C3	1.391 (10)	C50—H50A	0.9700
C3—H3	0.9300	C50—H50B	0.9700
C3—C4	1.371 (11)	C50—C51	1.540 (16)
C4—H4	0.9300	C62—H62A	0.9700
C4—C5	1.343 (10)	C62—H62B	0.9700
C5—H5	0.9300	C62—C61	1.519 (17)
C6—C7	1.396 (9)	C62—C63	1.527 (18)
C6—C11	1.382 (9)	C60—H60A	0.9700
C7—H7	0.9300	C60—H60B	0.9700
C7—C8	1.375 (10)	C60—C61	1.496 (17)
C8—H8	0.9300	C60—C59	1.498 (16)
C8—C9	1.397 (10)	C61—H61A	0.9700
C9—C10	1.377 (9)	C61—H61B	0.9700
C10—H10	0.9300	C64—H64A	0.9600
C10—C11	1.424 (10)	C64—H64B	0.9600
C12—H12A	0.9700	C64—H64C	0.9600
C12—H12B	0.9700	C64—C63	1.535 (17)
C12—C13	1.518 (10)	C63—H63A	0.9700
C13—H13A	0.9700	C63—H63B	0.9700
C13—H13B	0.9700	C58—H58A	0.9700
C13—C14	1.520 (10)	C58—H58B	0.9700
C14—H14A	0.9700	C58—C59	1.56 (3)
C14—H14B	0.9700	C58—C57	1.560 (17)
C14—C15	1.502 (11)	C59—H59A	0.9700
C15—H15A	0.9700	C59—H59B	0.9700
C15—H15B	0.9700	C56—H56A	0.9700
C16—C17	1.378 (11)	C56—H56B	0.9700
C16—C21	1.411 (10)	C56—C57	1.527 (16)
C17—H17	0.9300	C56—C55	1.627 (15)
C17—C18	1.384 (11)	C57—H57A	0.9700
C18—H18	0.9300	C57—H57B	0.9700
C18—C19	1.374 (10)	C51—H51A	0.9700
C19—C20	1.398 (11)	C51—H51B	0.9700
C19—C22	1.465 (11)	C51—C52	1.582 (15)
C20—H20	0.9300	C52—H52A	0.9700
C20—C21	1.363 (11)	C52—H52B	0.9700
C21—H21	0.9300	C52—C53	1.528 (15)
C22—C23	1.402 (13)	C54—H54A	0.9700
C22—C27	1.396 (11)	C54—H54B	0.9700
C23—H23	0.9300	C54—C53	1.582 (15)
C23—C24	1.362 (14)	C54—C55	1.500 (15)
C24—H24	0.9300	C53—H53A	0.9700
C24—C25	1.391 (15)	C53—H53B	0.9700
C25—C26	1.368 (14)	C55—H55A	0.9700
C25—C28	1.409 (16)	C55—H55B	0.9700
C26—H26	0.9300	C50A—H50C	0.9700
C26—C27	1.371 (13)	C50A—H50D	0.9700
C27—H27	0.9300	C50A—C51A	1.502 (18)
C29—H29A	0.9700	C51A—H51C	0.9700
C29—H29B	0.9700	C51A—H51D	0.9700
C29—C30	1.537 (12)	C51A—C52A	1.590 (17)
C30—H30A	0.9700	C52A—H52C	0.9700
C30—H30B	0.9700	C52A—H52D	0.9700
C30—C31	1.441 (12)	C52A—C53A	1.636 (17)
C31—H31A	0.9700	C53A—H53C	0.9700
C31—H31B	0.9700	C53A—H53D	0.9700
C31—C32	1.582 (14)	C53A—C54A	1.584 (17)
C32—H32A	0.9700	C54A—H54C	0.9700
C32—H32B	0.9700	C54A—H54D	0.9700
C32—C33	1.450 (14)	C54A—C55A	1.537 (17)
C33—H33A	0.9700	C55A—H55C	0.9700
C33—H33B	0.9700	C55A—H55D	0.9700
C33—C34	1.507 (13)	C55A—C56A	1.584 (18)
C34—H34A	0.9700	C56A—H56C	0.9700
C34—H34B	0.9700	C56A—H56D	0.9700
C34—C35	1.473 (14)	C56A—C57A	1.525 (18)
C35—H35A	0.9700	C57A—H57C	0.9700
C35—H35B	0.9700	C57A—H57D	0.9700
C35—C36	1.454 (13)	C57A—C58A	1.562 (19)
C36—H36A	0.9700	C58A—H58C	0.9700
C36—H36B	0.9700	C58A—H58D	0.9700
C36—C37	1.482 (13)	C58A—C59A	1.54 (2)
C37—H37A	0.9700	C59A—H59C	0.9700
C37—H37B	0.9700	C59A—H59D	0.9700
C37—C38	1.472 (14)	C59A—C60A	1.525 (19)
C38—H38A	0.9700	C60A—H60C	0.9700
C38—H38B	0.9700	C60A—H60D	0.9700
C38—C39	1.478 (14)	C60A—C61A	1.517 (19)
C39—H39A	0.9700	C61A—H61C	0.9700
C39—H39B	0.9700	C61A—H61D	0.9700
C39—C40	1.470 (15)	C61A—C62A	1.534 (19)
C40—H40A	0.9600	C62A—H62C	0.9700
C40—H40B	0.9600	C62A—H62D	0.9700
C40—H40C	0.9600	C62A—C63A	1.533 (19)
C41—C42	1.364 (12)	C63A—H63C	0.9700
C41—C44	1.529 (12)	C63A—H63D	0.9700
C42—H42	0.9300	C63A—C64A	1.539 (18)
C42—C43	1.380 (14)	C64A—H64D	0.9600
C43—C50	1.588 (15)	C64A—H64E	0.9600
C43—C50A	1.495 (17)	C64A—H64F	0.9600
C44—H44A	0.9700		
			
O1—Pt1—O2	91.1 (3)	H45A—C45—H45B	107.8
N1—Pt1—O1	174.2 (2)	C46—C45—H45A	109.0
N1—Pt1—O2	94.6 (3)	C46—C45—H45B	109.0
C11—Pt1—O1	92.7 (2)	C45—C46—H46A	108.9
C11—Pt1—O2	176.1 (3)	C45—C46—H46B	108.9
C11—Pt1—N1	81.6 (3)	H46A—C46—H46B	107.7
C41—O1—Pt1	124.8 (6)	C47—C46—C45	113.4 (7)
C43—O2—Pt1	124.7 (8)	C47—C46—H46A	108.9
C2—O3—C12	119.1 (6)	C47—C46—H46B	108.9
C16—O4—C15	116.6 (6)	C46—C47—H47A	108.3
C9—O5—C29	118.5 (6)	C46—C47—H47B	108.3
C1—N1—Pt1	115.5 (5)	H47A—C47—H47B	107.4
C5—N1—Pt1	125.2 (5)	C48—C47—C46	116.0 (9)
C5—N1—C1	119.3 (6)	C48—C47—H47A	108.3
N1—C1—C2	118.8 (6)	C48—C47—H47B	108.3
N1—C1—C6	113.5 (6)	C47—C48—H48A	108.3
C2—C1—C6	127.7 (6)	C47—C48—H48B	108.3
O3—C2—C1	116.3 (6)	H48A—C48—H48B	107.4
O3—C2—C3	123.9 (7)	C49—C48—C47	116.0 (11)
C3—C2—C1	119.8 (7)	C49—C48—H48A	108.3
C2—C3—H3	120.5	C49—C48—H48B	108.3
C4—C3—C2	118.9 (8)	C48—C49—H49A	109.5
C4—C3—H3	120.5	C48—C49—H49B	109.5
C3—C4—H4	119.9	C48—C49—H49C	109.5
C5—C4—C3	120.2 (8)	H49A—C49—H49B	109.5
C5—C4—H4	119.9	H49A—C49—H49C	109.5
N1—C5—H5	118.5	H49B—C49—H49C	109.5
C4—C5—N1	123.0 (7)	C43—C50—H50A	110.3
C4—C5—H5	118.5	C43—C50—H50B	110.3
C7—C6—C1	124.7 (6)	H50A—C50—H50B	108.6
C11—C6—C1	112.7 (6)	C51—C50—C43	107.0 (12)
C11—C6—C7	122.6 (7)	C51—C50—H50A	110.3
C6—C7—H7	121.1	C51—C50—H50B	110.3
C8—C7—C6	117.8 (6)	H62A—C62—H62B	107.7
C8—C7—H7	121.1	C61—C62—H62A	108.9
C7—C8—H8	119.1	C61—C62—H62B	108.9
C7—C8—C9	121.8 (7)	C61—C62—C63	113 (2)
C9—C8—H8	119.1	C63—C62—H62A	108.9
O5—C9—C8	115.9 (7)	C63—C62—H62B	108.9
O5—C9—C10	124.4 (7)	H60A—C60—H60B	106.0
C10—C9—C8	119.7 (7)	C61—C60—H60A	105.4
C9—C10—H10	119.9	C61—C60—H60B	105.4
C9—C10—C11	120.1 (6)	C61—C60—C59	128 (2)
C11—C10—H10	119.9	C59—C60—H60A	105.4
C6—C11—Pt1	116.6 (6)	C59—C60—H60B	105.4
C6—C11—C10	118.0 (7)	C62—C61—H61A	107.3
C10—C11—Pt1	125.4 (5)	C62—C61—H61B	107.3
O3—C12—H12A	110.7	C60—C61—C62	120 (2)
O3—C12—H12B	110.7	C60—C61—H61A	107.3
O3—C12—C13	105.2 (6)	C60—C61—H61B	107.3
H12A—C12—H12B	108.8	H61A—C61—H61B	106.9
C13—C12—H12A	110.7	C62—C63—C64	103.5 (18)
C13—C12—H12B	110.7	C62—C63—H63A	111.1
C12—C13—H13A	109.0	C62—C63—H63B	111.1
C12—C13—H13B	109.0	C64—C63—H63A	111.1
C12—C13—C14	112.7 (7)	C64—C63—H63B	111.1
H13A—C13—H13B	107.8	H63A—C63—H63B	109.0
C14—C13—H13A	109.0	H58A—C58—H58B	108.9
C14—C13—H13B	109.0	C59—C58—H58A	110.9
C13—C14—H14A	108.9	C59—C58—H58B	110.9
C13—C14—H14B	108.9	C59—C58—C57	104.3 (17)
H14A—C14—H14B	107.8	C57—C58—H58A	110.9
C15—C14—C13	113.2 (7)	C57—C58—H58B	110.9
C15—C14—H14A	108.9	C60—C59—C58	115.8 (18)
C15—C14—H14B	108.9	C60—C59—H59A	108.3
O4—C15—C14	108.5 (7)	C60—C59—H59B	108.3
O4—C15—H15A	110.0	C58—C59—H59A	108.3
O4—C15—H15B	110.0	C58—C59—H59B	108.3
C14—C15—H15A	110.0	H59A—C59—H59B	107.4
C14—C15—H15B	110.0	H56A—C56—H56B	108.3
H15A—C15—H15B	108.4	C57—C56—H56A	109.9
O4—C16—C17	124.7 (7)	C57—C56—H56B	109.9
O4—C16—C21	115.8 (8)	C57—C56—C55	108.8 (13)
C17—C16—C21	119.5 (8)	C55—C56—H56A	109.9
C16—C17—H17	120.3	C55—C56—H56B	109.9
C16—C17—C18	119.4 (8)	C58—C57—H57A	110.3
C18—C17—H17	120.3	C58—C57—H57B	110.3
C17—C18—H18	118.7	C56—C57—C58	107.3 (15)
C19—C18—C17	122.7 (8)	C56—C57—H57A	110.3
C19—C18—H18	118.7	C56—C57—H57B	110.3
C18—C19—C20	116.5 (8)	H57A—C57—H57B	108.5
C18—C19—C22	121.2 (8)	C50—C51—H51A	108.5
C20—C19—C22	122.2 (7)	C50—C51—H51B	108.5
C19—C20—H20	118.5	C50—C51—C52	114.9 (13)
C21—C20—C19	122.9 (7)	H51A—C51—H51B	107.5
C21—C20—H20	118.5	C52—C51—H51A	108.5
C16—C21—H21	120.6	C52—C51—H51B	108.5
C20—C21—C16	118.8 (8)	C51—C52—H52A	111.1
C20—C21—H21	120.6	C51—C52—H52B	111.1
C23—C22—C19	121.3 (8)	H52A—C52—H52B	109.1
C27—C22—C19	122.8 (8)	C53—C52—C51	103.3 (13)
C27—C22—C23	116.0 (9)	C53—C52—H52A	111.1
C22—C23—H23	119.0	C53—C52—H52B	111.1
C24—C23—C22	122.1 (10)	H54A—C54—H54B	107.4
C24—C23—H23	119.0	C53—C54—H54A	108.3
C23—C24—H24	120.3	C53—C54—H54B	108.3
C23—C24—C25	119.5 (11)	C55—C54—H54A	108.3
C25—C24—H24	120.3	C55—C54—H54B	108.3
C24—C25—C28	116.9 (13)	C55—C54—C53	115.8 (14)
C26—C25—C24	120.6 (11)	C52—C53—C54	108.8 (13)
C26—C25—C28	122.5 (12)	C52—C53—H53A	109.9
C25—C26—H26	120.5	C52—C53—H53B	109.9
C25—C26—C27	118.9 (9)	C54—C53—H53A	109.9
C27—C26—H26	120.5	C54—C53—H53B	109.9
C22—C27—H27	118.5	H53A—C53—H53B	108.3
C26—C27—C22	122.9 (9)	C56—C55—H55A	108.5
C26—C27—H27	118.5	C56—C55—H55B	108.5
N2—C28—C25	177 (2)	C54—C55—C56	114.9 (13)
O5—C29—H29A	110.3	C54—C55—H55A	108.5
O5—C29—H29B	110.3	C54—C55—H55B	108.5
O5—C29—C30	107.3 (7)	H55A—C55—H55B	107.5
H29A—C29—H29B	108.5	C43—C50A—H50C	110.2
C30—C29—H29A	110.3	C43—C50A—H50D	110.2
C30—C29—H29B	110.3	C43—C50A—C51A	107.4 (17)
C29—C30—H30A	109.3	H50C—C50A—H50D	108.5
C29—C30—H30B	109.3	C51A—C50A—H50C	110.2
H30A—C30—H30B	107.9	C51A—C50A—H50D	110.2
C31—C30—C29	111.7 (9)	C50A—C51A—H51C	111.0
C31—C30—H30A	109.3	C50A—C51A—H51D	111.0
C31—C30—H30B	109.3	C50A—C51A—C52A	103.7 (18)
C30—C31—H31A	109.5	H51C—C51A—H51D	109.0
C30—C31—H31B	109.5	C52A—C51A—H51C	111.0
C30—C31—C32	110.5 (10)	C52A—C51A—H51D	111.0
H31A—C31—H31B	108.1	C51A—C52A—H52C	107.4
C32—C31—H31A	109.5	C51A—C52A—H52D	107.4
C32—C31—H31B	109.5	C51A—C52A—C53A	119.8 (19)
C31—C32—H32A	108.9	H52C—C52A—H52D	106.9
C31—C32—H32B	108.9	C53A—C52A—H52C	107.4
H32A—C32—H32B	107.7	C53A—C52A—H52D	107.4
C33—C32—C31	113.6 (10)	C52A—C53A—H53C	104.8
C33—C32—H32A	108.9	C52A—C53A—H53D	104.8
C33—C32—H32B	108.9	H53C—C53A—H53D	105.8
C32—C33—H33A	107.9	C54A—C53A—C52A	129.8 (19)
C32—C33—H33B	107.9	C54A—C53A—H53C	104.8
C32—C33—C34	117.8 (11)	C54A—C53A—H53D	104.8
H33A—C33—H33B	107.2	C53A—C54A—H54C	107.8
C34—C33—H33A	107.9	C53A—C54A—H54D	107.8
C34—C33—H33B	107.9	H54C—C54A—H54D	107.1
C33—C34—H34A	108.1	C55A—C54A—C53A	118 (2)
C33—C34—H34B	108.1	C55A—C54A—H54C	107.8
H34A—C34—H34B	107.3	C55A—C54A—H54D	107.8
C35—C34—C33	117.0 (10)	C54A—C55A—H55C	108.0
C35—C34—H34A	108.1	C54A—C55A—H55D	108.0
C35—C34—H34B	108.1	C54A—C55A—C56A	117 (2)
C34—C35—H35A	107.5	H55C—C55A—H55D	107.2
C34—C35—H35B	107.5	C56A—C55A—H55C	108.0
H35A—C35—H35B	107.0	C56A—C55A—H55D	108.0
C36—C35—C34	119.4 (10)	C55A—C56A—H56C	106.3
C36—C35—H35A	107.5	C55A—C56A—H56D	106.3
C36—C35—H35B	107.5	H56C—C56A—H56D	106.4
C35—C36—H36A	107.5	C57A—C56A—C55A	124 (2)
C35—C36—H36B	107.5	C57A—C56A—H56C	106.3
C35—C36—C37	119.4 (10)	C57A—C56A—H56D	106.3
H36A—C36—H36B	107.0	C56A—C57A—H57C	104.4
C37—C36—H36A	107.5	C56A—C57A—H57D	104.4
C37—C36—H36B	107.5	C56A—C57A—C58A	131 (2)
C36—C37—H37A	108.1	H57C—C57A—H57D	105.6
C36—C37—H37B	108.1	C58A—C57A—H57C	104.4
H37A—C37—H37B	107.3	C58A—C57A—H57D	104.4
C38—C37—C36	116.8 (10)	C57A—C58A—H58C	108.0
C38—C37—H37A	108.1	C57A—C58A—H58D	108.0
C38—C37—H37B	108.1	H58C—C58A—H58D	107.3
C37—C38—H38A	108.2	C59A—C58A—C57A	117 (3)
C37—C38—H38B	108.2	C59A—C58A—H58C	108.0
C37—C38—C39	116.2 (11)	C59A—C58A—H58D	108.0
H38A—C38—H38B	107.4	C58A—C59A—H59C	108.8
C39—C38—H38A	108.2	C58A—C59A—H59D	108.8
C39—C38—H38B	108.2	H59C—C59A—H59D	107.7
C38—C39—H39A	108.2	C60A—C59A—C58A	114 (3)
C38—C39—H39B	108.2	C60A—C59A—H59C	108.8
H39A—C39—H39B	107.4	C60A—C59A—H59D	108.8
C40—C39—C38	116.2 (12)	C59A—C60A—H60C	110.6
C40—C39—H39A	108.2	C59A—C60A—H60D	110.6
C40—C39—H39B	108.2	H60C—C60A—H60D	108.7
C39—C40—H40A	109.5	C61A—C60A—C59A	106 (3)
C39—C40—H40B	109.5	C61A—C60A—H60C	110.6
C39—C40—H40C	109.5	C61A—C60A—H60D	110.6
H40A—C40—H40B	109.5	C60A—C61A—H61C	110.3
H40A—C40—H40C	109.5	C60A—C61A—H61D	110.3
H40B—C40—H40C	109.5	C60A—C61A—C62A	107 (3)
O1—C41—C42	126.2 (9)	H61C—C61A—H61D	108.6
O1—C41—C44	111.9 (8)	C62A—C61A—H61C	110.3
C42—C41—C44	121.9 (8)	C62A—C61A—H61D	110.3
C41—C42—H42	115.5	C61A—C62A—H62C	109.7
C41—C42—C43	128.9 (9)	C61A—C62A—H62D	109.7
C43—C42—H42	115.5	H62C—C62A—H62D	108.2
O2—C43—C42	124.2 (10)	C63A—C62A—C61A	110 (3)
O2—C43—C50	108.5 (13)	C63A—C62A—H62C	109.7
O2—C43—C50A	127.2 (15)	C63A—C62A—H62D	109.7
C42—C43—C50	127.3 (13)	C62A—C63A—H63C	105.4
C42—C43—C50A	108.6 (14)	C62A—C63A—H63D	105.4
C50A—C43—C50	18.9 (15)	C62A—C63A—C64A	128 (3)
C41—C44—H44A	108.1	H63C—C63A—H63D	106.0
C41—C44—H44B	108.1	C64A—C63A—H63C	105.4
H44A—C44—H44B	107.3	C64A—C63A—H63D	105.4
C45—C44—C41	116.8 (7)	C63A—C64A—H64D	109.5
C45—C44—H44A	108.1	C63A—C64A—H64E	109.5
C45—C44—H44B	108.1	C63A—C64A—H64F	109.5
C44—C45—H45A	109.0	H64D—C64A—H64E	109.5
C44—C45—H45B	109.0	H64D—C64A—H64F	109.5
C44—C45—C46	113.1 (7)	H64E—C64A—H64F	109.5
			
Pt1—O1—C41—C42	2.6 (10)	C17—C18—C19—C20	−3.4 (14)
Pt1—O1—C41—C44	−177.1 (4)	C17—C18—C19—C22	175.5 (9)
Pt1—O2—C43—C42	−2.3 (15)	C18—C19—C20—C21	2.0 (13)
Pt1—O2—C43—C50	176.2 (8)	C18—C19—C22—C23	35.3 (14)
Pt1—O2—C43—C50A	178.8 (15)	C18—C19—C22—C27	−144.8 (9)
Pt1—N1—C1—C2	−179.1 (4)	C19—C20—C21—C16	−0.5 (13)
Pt1—N1—C1—C6	−0.5 (6)	C19—C22—C23—C24	−177.4 (11)
Pt1—N1—C5—C4	177.1 (5)	C19—C22—C27—C26	178.6 (8)
O1—Pt1—O2—C43	4.3 (8)	C20—C19—C22—C23	−145.8 (10)
O1—Pt1—N1—C1	12.4 (19)	C20—C19—C22—C27	34.1 (13)
O1—Pt1—N1—C5	−164.8 (15)	C21—C16—C17—C18	−1.6 (14)
O1—Pt1—C11—C6	177.5 (4)	C22—C19—C20—C21	−176.9 (8)
O1—Pt1—C11—C10	−0.4 (5)	C22—C23—C24—C25	−2 (2)
O1—C41—C42—C43	1.3 (14)	C23—C22—C27—C26	−1.6 (14)
O1—C41—C44—C45	−1.5 (9)	C23—C24—C25—C26	−1 (2)
O2—Pt1—O1—C41	−4.3 (5)	C23—C24—C25—C28	−178.9 (14)
O2—Pt1—N1—C1	−176.7 (4)	C24—C25—C26—C27	2.2 (19)
O2—Pt1—N1—C5	6.1 (5)	C24—C25—C28—N2	−134 (31)
O2—Pt1—C11—C6	14 (3)	C25—C26—C27—C22	−0.8 (15)
O2—Pt1—C11—C10	−164 (3)	C26—C25—C28—N2	48 (32)
O2—C43—C50—C51	108.5 (16)	C27—C22—C23—C24	2.7 (17)
O2—C43—C50A—C51A	36 (3)	C28—C25—C26—C27	180.0 (13)
O3—C2—C3—C4	177.9 (6)	C29—O5—C9—C8	178.2 (7)
O3—C12—C13—C14	−177.8 (6)	C29—O5—C9—C10	−3.4 (11)
O4—C16—C17—C18	−178.4 (8)	C29—C30—C31—C32	169.2 (9)
O4—C16—C21—C20	177.3 (7)	C30—C31—C32—C33	−171.2 (11)
O5—C9—C10—C11	179.9 (6)	C31—C32—C33—C34	176.4 (11)
O5—C29—C30—C31	−65.6 (11)	C32—C33—C34—C35	−176.7 (12)
N1—Pt1—O1—C41	166.6 (15)	C33—C34—C35—C36	177.3 (12)
N1—Pt1—O2—C43	−174.8 (8)	C34—C35—C36—C37	179.1 (12)
N1—Pt1—C11—C6	−3.5 (4)	C35—C36—C37—C38	179.0 (12)
N1—Pt1—C11—C10	178.5 (5)	C36—C37—C38—C39	−178.6 (12)
N1—C1—C2—O3	−176.7 (5)	C37—C38—C39—C40	−178.9 (13)
N1—C1—C2—C3	2.0 (9)	C41—C42—C43—O2	−1.4 (17)
N1—C1—C6—C7	−179.9 (6)	C41—C42—C43—C50	−179.6 (11)
N1—C1—C6—C11	−2.3 (7)	C41—C42—C43—C50A	177.7 (14)
C1—N1—C5—C4	0.0 (10)	C41—C44—C45—C46	−179.1 (6)
C1—C2—C3—C4	−0.6 (10)	C42—C41—C44—C45	178.9 (7)
C1—C6—C7—C8	177.0 (6)	C42—C43—C50—C51	−73 (2)
C1—C6—C11—Pt1	4.1 (7)	C42—C43—C50A—C51A	−143 (2)
C1—C6—C11—C10	−177.8 (5)	C43—C50—C51—C52	−172.3 (14)
C2—O3—C12—C13	179.3 (6)	C43—C50A—C51A—C52A	−174.9 (18)
C2—C1—C6—C7	−1.5 (10)	C44—C41—C42—C43	−179.1 (9)
C2—C1—C6—C11	176.1 (6)	C44—C45—C46—C47	−176.9 (6)
C2—C3—C4—C5	−1.0 (11)	C45—C46—C47—C48	179.8 (7)
C3—C4—C5—N1	1.4 (11)	C46—C47—C48—C49	179.4 (10)
C5—N1—C1—C2	−1.7 (8)	C50—C43—C50A—C51A	44 (3)
C5—N1—C1—C6	176.9 (5)	C50—C51—C52—C53	−65 (2)
C6—C1—C2—O3	5.0 (9)	C61—C62—C63—C64	176 (2)
C6—C1—C2—C3	−176.3 (6)	C61—C60—C59—C58	−141 (3)
C6—C7—C8—C9	−0.1 (10)	C63—C62—C61—C60	−175 (3)
C7—C6—C11—Pt1	−178.2 (5)	C59—C60—C61—C62	−171 (3)
C7—C6—C11—C10	−0.1 (9)	C59—C58—C57—C56	172.0 (17)
C7—C8—C9—O5	179.7 (7)	C57—C58—C59—C60	−170 (2)
C7—C8—C9—C10	1.3 (11)	C57—C56—C55—C54	−176.0 (16)
C8—C9—C10—C11	−1.8 (10)	C51—C52—C53—C54	−166.8 (13)
C9—O5—C29—C30	−171.8 (7)	C53—C54—C55—C56	−153.2 (16)
C9—C10—C11—Pt1	179.2 (5)	C55—C56—C57—C58	142.3 (19)
C9—C10—C11—C6	1.2 (9)	C55—C54—C53—C52	166.8 (16)
C11—Pt1—O1—C41	176.8 (5)	C50A—C43—C50—C51	−65 (4)
C11—Pt1—O2—C43	168 (3)	C50A—C51A—C52A—C53A	−79 (3)
C11—Pt1—N1—C1	2.1 (4)	C51A—C52A—C53A—C54A	−162 (2)
C11—Pt1—N1—C5	−175.1 (5)	C52A—C53A—C54A—C55A	−103 (3)
C11—C6—C7—C8	−0.4 (10)	C53A—C54A—C55A—C56A	132 (2)
C12—O3—C2—C1	−177.1 (5)	C54A—C55A—C56A—C57A	172 (2)
C12—O3—C2—C3	4.3 (9)	C55A—C56A—C57A—C58A	−99 (4)
C12—C13—C14—C15	169.2 (7)	C56A—C57A—C58A—C59A	−149 (3)
C13—C14—C15—O4	73.5 (8)	C57A—C58A—C59A—C60A	161 (3)
C15—O4—C16—C17	12.0 (12)	C58A—C59A—C60A—C61A	155 (4)
C15—O4—C16—C21	−164.9 (7)	C59A—C60A—C61A—C62A	152 (4)
C16—O4—C15—C14	175.3 (7)	C60A—C61A—C62A—C63A	176 (4)
C16—C17—C18—C19	3.3 (15)	C61A—C62A—C63A—C64A	173 (3)
C17—C16—C21—C20	0.2 (12)		
