# Supplementary figures and images for: Crystal structure of (2-{3-[4-(4′-cyano­biphenyl-4-yl­oxy)but­oxy]pyridin-2-yl-κN}-5-(dodec­yloxy)phenyl-κC 1)(9-oxo­tetra­cos-7-en-7-olato-κ2 O,O′)platinum(II)
Source: Acta Crystallogr Sect E Struct Rep Online. 2014 Nov 5;70(Pt 12):m392–3. doi: 10.1107/S1600536814021928 (PMC4257408; doi:10.1107/S1600536814021928)

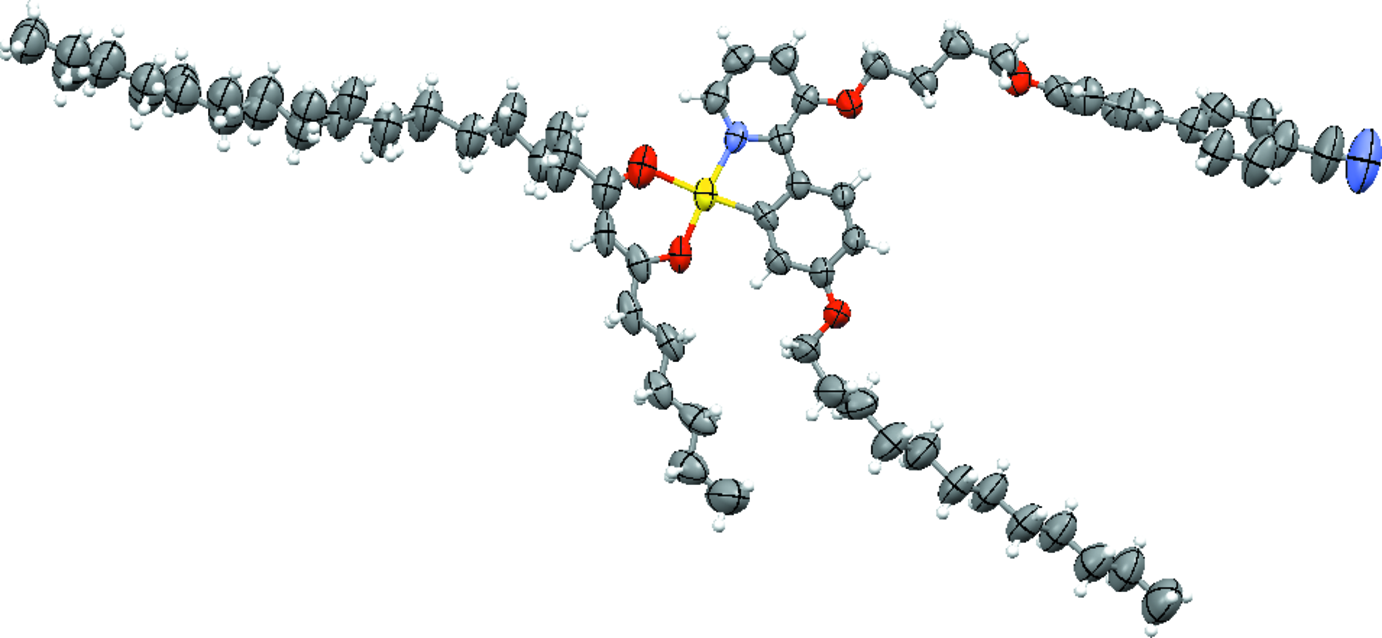

Supplement: Supplementary file 3 [file e-70-0m392-fig1.tif]
